# Developing an Impedance Based Equation for Fat-Free Mass of Black Preadolescent South African Children

**DOI:** 10.3390/nu11092021

**Published:** 2019-08-28

**Authors:** Amanda van Zyl, Zelda White, Johan Ferreira, Friedeburg A. M. Wenhold

**Affiliations:** 1 Department Human Nutrition, Faculty of Health Sciences, University of Pretoria, Private Bag X323, Arcadia 0007, South Africa; 2 Department of Statistics, Faculty of Natural and Agricultural Sciences, University of Pretoria, Private Bag X20, Hatfield 0028, South Africa

**Keywords:** bioelectrical impedance analysis (BIA), body composition, fat-free mass (FFM), dual energy X-ray absorptiometry (DXA), preadolescent, prediction equations

## Abstract

Bioelectrical impedance analysis (BIA) is a practical alternative to dual-energy X-ray absorptiometry (DXA) for determining body composition in children. Currently, there are no population specific equations available for predicting fat-free mass (FFM) in South African populations. We determined agreement between fat-free mass measured by DXA (FFM_DXA_) and FFM calculated from published multi-frequency bioelectrical impedance prediction equations (FFM_BIA_); and developed a new equation for predicting FFM for preadolescent black South African children. Cross-sectional data on a convenience sample of 84 children (mean age 8.5 ± 1.4 years; 44 {52%} girls) included body composition assessed using Dual X-ray Absorptiometry (FFM_DXA_) and impedance values obtained from the Seca mBCA 514 Medical Body Composition analyzer used to calculate FFM using 17 published prediction equations (FFM_BIA_). Only two equations yielded FFM estimates that were similar to the DXA readings (*p* > 0.05). According to the Bland–Altman analysis, the mean differences in FFM (kg) were 0.15 (LOA: −2.68; 2.37) and 0.01 (LOA: −2.68; 2.66). Our new prediction equation, FFM=105.20+0.807×Sex+0.174×Weight+0.01×Reactance+15.71×log(RI), yielded an adjusted R^2^ = 0.9544. No statistical shrinkage was observed during cross-validation. A new equation enables the BIA-based prediction of FFM in the assessment of preadolescent black South African children.

## 1. Introduction

The prevalence of childhood obesity in industrialized and low-middle income countries is increasing and constitutes a major public health hazard [1]. The South Africa Demographic and Health Survey showed that 13% of children younger than five years were classified as overweight [2]. Overweight and obesity in infancy and childhood may be risk factors for developing non-communicable diseases (NCDs) later in life [3]. In South Africa, as in other low-middle income countries, the burden of disease is shifting from communicable diseases to NCDs [4,5]. Since obesity is an important risk factor for many NCDs, there is an increasing demand for accurate body composition assessments. 

Body composition assessments estimate the proportions of fat mass (FM) and fat-free mass (FFM). Fat mass includes fat from brain tissue, the skeleton as well as adipose tissue, and FFM comprises the water, protein and mineral components of the body. Body composition assessments also measure fat distribution and body water (intra-cellular and extra-cellular) [6]. Body composition measurements help to monitor the risk of developing NCDs since the proportion and distribution of body fat is a major component of metabolic load [7]. Obesity is often measured in children using simple metrics, including height, weight, and body mass index (BMI) for age, but these methods do not distinguish between FM and FFM and cannot measure changes in body composition. Simple metrics may also conceal deficits in FFM by focusing on excess FM [8]. Using body composition assessments, health care professionals will be able to identify children with excessive FM at an early age, creating an opportunity for health promotion and targeted prevention of NCDs [9]. 

Several direct and indirect methods are available to determine body composition. These methods differ in terms of availability, cost, accuracy, exposure to radiation, and invasiveness. Dual energy X-ray absorptiometry (DXA) is considered to be the reference method for determining body composition, as it is accurate, precise, and suitable for children due to minimal radiation exposure and relative comfort. Dual energy X-ray absorptiometry is costly, time consuming, requires technical expertise and is not always readily available [9]. Bioelectrical impedance analysis (BIA) offers a practical alternative, but its validity is threatened by assumptions related to body shape and hydration [10]. 

Bioelectrical impedance analysis is increasingly used to determine body composition of children. Bioelectrical impedance analysis is an indirect method, whereby the human body reacts to a small electrical current by offering resistance. The recorded impedance values (the resistance *[R]* and reactance *[Xc]*) are applied to equations predicting estimates of body composition [11]. The prediction equations used in BIA have been derived from linear regressions where the response variable is obtained using a reference method such as DXA [9]. In the literature, BIA prediction equations mostly represent adult populations, and data representing children of different ages and ethnicities are still limited [12,13].

Currently, different BIA devices, using different techniques, are available [11]. A more recent and sophisticated BIA technique, multi-frequency BIA and segmental BIA, improves the accuracy of body composition assessments by allowing for variations in body build [14,15]. More recently, the Seca mBCA 514 Medical Body Composition analyzer has been validated for use in healthy adults [10,16,17] with limited applicability for children [18] and, more specifically, for black South African children. In this study, we measure the body composition of black South African preadolescent children using DXA as a reference method and compare these values to estimates of body composition derived from published predication equations for multi-frequency BIA. We determined the agreement between FFM measured by DXA and BIA prediction equations. We also developed a population specific equation for predicting FFM of preadolescent, black South African children.

## 2. Materials and Methods 

### 2.1. Participants and Setting

In this cross-sectional study, we recruited a convenience sample of black children (self-reported race/ethnicity) from two after-school care centers at a primary school in Arcadia, Pretoria, South Africa. As a precautionary measure, we excluded children with physical disabilities, electrical and metallic implants (e.g., pacemakers) or those who were connected to portable electronic devices. The protocol was approved by the Research Ethics Committee, Faculty of Health Sciences, University of Pretoria (No: 73/2016). Parental consent and child assent were obtained from each participant.

Participants were instructed to avoid eating and drinking two hours before the measurements and were given a standard meal of 250g drinking yogurt while being transported to the radiology department were measurements were taken. Participants were asked to void their bladder and change into hospital gowns before the assessment. All measurements were taken on the same day within half an hour for each participant.

### 2.2. Anthropometric Measurements 

We measured standing height, to the nearest 0.1 cm, using a calibrated wireless Seca 274 stadiometer (Hamburg, Germany). We weighed each participant to the nearest 100 g, using the calibrated Seca mBCA 514 (version 1.4.292.4928, Hamburg, Germany). We described the anthropometric status of each participant using *z* scores for height-for-age (HFA*z*) and BMI-for-age (BMIFA*z*) based on the World Health Organization (WHO) reference values for school aged children [19].

### 2.3. DXA Measurement 

We performed whole-body DXA scans on each participant in the standard supine position, using the Hologic Discovery W densitometer (v13.4.2 software) (Hologic Discovery, Hologic Inc., Bedford, MA, USA). The DXA scans provided reference values of fat-free mass (FFM_DXA_), representing lean mass + bone mineral content (total body less the head). 

### 2.4. Fat-Free Mass Calculations from BIA (FFM_BIA_)

We used the multi-frequency Seca mBCA 514 Medical Body Composition analyzer to measure raw impedance data (reactance *[R]* and resistance *[Xc]*, measured at 50 kHz) of each participant. Impedance (Z) was calculated as [20]:

$$Z=\surd \left(R^{2}+X^{2}\right)$$

We also calculated the resistance index $\left(RI=height^{2}/R\right)$ (cm^2^/Ω) and impedance index $\left(ZI=height^{2}/R\right)$ (cm^2^/Ω) for use in prediction equations.

The calculated impedance values were fed into 17 BIA-based prediction equations identified in the literature that met the following inclusion criteria: (1) developed for both boys and girls aged 5- to 10-years old; (2) requiring at least one of the following equation parameters: basic anthropometric measurements (height and/or weight), age, sex, race/ethnicity, and resistance or impedance values; and (3) comparison of BIA to a reference method (correlation-, agreement- or validation studies).

We used the Student’s two sample *t*-test to test for differences in descriptive characteristics and body composition estimates between boys and girls. Results were significant if *p* ≤ 0.05. 

### 2.5. Agreement between FFM_DXA_ and FFM_BIA_

We measured agreement between the FFM_DXA_ and FFM_BIA_ values using STATA version 14.2 software. The population accuracy of the predictive equations was calculated using the mean bias, with a reliable estimate defined as a mean percentage difference ±5% between FFM_BIA_ and FFM_DXA_. The percentage of individuals with a FFM_BIA_-FFM_DXA_ difference within ±5% was considered as a measure of accuracy at an individual level. Values lower than 95% indicated underprediction and values higher than 105% overprediction [21].

We quantified the differences between FFM_DXA_ and FFM_BIA_ using the paired *t*-test, Bland–Altman [22] plot analyses and intra-class correlation coefficients (ICC). Prediction equations were deemed suitable for this population if mean FFM_DXA_ and FFM_BIA_ were statistically similar (*p* > 0.05), the differences in mean values were close to 0, high intra-class correlation (ICC) (close to 1) and small limits of agreement (LoA) (<15% ½ interval width). The acceptable LoA was selected in context, based on a statistically acceptable value [23].

We conducted a Bland–Altman plot analysis for each equation that gave FFM_BIA_ values similar to FFM_DXA_ values. The differences in FFM (kg) $\left(\Delta FFM=FFM_{DXA}-FFM_{BIA}\right)$ were plotted against mean FFM_BIA_ and FFM_DXA_ values. Limits of agreement (LoA) were calculated using the mean and SD of the differences (ΔFFM). We also conducted a power analysis between FFM_BIA_ and FFM_DXA_ values. The power analysis entailed determining the power of the test, using proc power in SAS 9.4 (SAS Institute Inc., Cary, NC, USA). The power analysis was included to illustrate how the data allowed the practitioner to pick up departures from the null hypothesis of equality.

### 2.6. Developing a New BIA Equation

We constructed a BIA equation using multiple regression analyses. The regression model was built using PROC REG, SAS Software 9.4 (code available on request). In the regression model, FFM_DXA_ was the response variable, and predictor variables included weight, reactance value, sex, resistance index, and impedance index. These variables have historically been included in BIA prediction equations. Starting with a full model, we investigated which predictor variables influenced the response variable, FFM_DXA._ Non-significant variables were deleted in a stepwise fashion until the final model included only significant variables. The optimal model was chosen based on high R^2^ values and small values of Mallows C. A summary of the stepwise selection is shown in Table 1, where “heightvar2” is equal to the logarithm of height squared divided by resistance 50 khz value (thus, log (RI)). It is clearly seen that the model R-square increased with the addition of the above variables. Other variables such as age were not considered a meaningful addition to the model, judged by the C(p) as well as the *p*-value for the F test under consideration for inclusion.

We tested if the residuals were normally distributed using the Kolmogorov–Smirnov, Cramer–von Mises, and Anderson–Darling tests for normality at a significance level of 95% (*p* < 0.05).

The new BIA equation was validated through correlation analysis of predicted FFM using the new BIA equation and the measured FFM_DXA_ of the participants. We used a *t*-test to test for differences between predicted FFM and FFM_DXA_.

## 3. Results

Our data analysis included 84 preadolescent children (mean age 8.5 ± 1.4 years; 44 (52%) girls). The characteristics of participants are presented in Table 2. The mean FFM_DXA_ (kg) of participants was 21.5 ± 4.9 kg. Boys had a significantly higher FFM_DXA_ than girls (*p* = 0.025). Girls had significantly higher mean percentage body fat than boys (*p* < 0.001). Thirty-four (41%) of participants had a BMIFAz >1.

### 3.1. Agreement between FFM_DXA_ and FFM_BIA_

**Figure 1:**
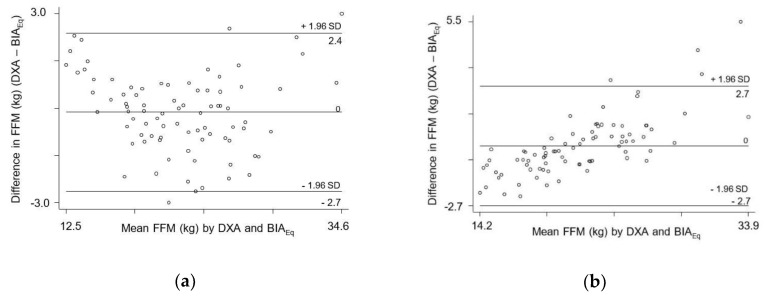
Bland–Altman plot analysis to evaluate the agreement between FFM_DXA_ and FFM_BIA._ (**a**) Clasey et al. [24] (**b**) Nielsen et al. [26].

Agreement between FFM_DXA_ and FFM calculated from BIA equations is presented in Table 3. Fat-free mass calculated from all equations had strong ICCs with FFM_DXA_. Three BIA equations [24,25,26] yielded FFM estimates similar to FFM_DXA_ (*p* = 0.269, *p* = 0.680 and *p* = 0.954). The power analysis agreed closely with expected results, showing a low power for each of these three equations. The Bland–Altman plots (Figure 1) indicated that two of these equations had acceptable LoA of –2.7 to 2.4 (Figure 1a) [24] and −2.7 to 2.7 (Figure 1b) [26]. Although these two equations yielded statistically acceptable LoA, these LoA might not be clinically acceptable, so we developed a new equation specific to this population. Five of the equations [27,28,29,30,31] significantly underestimated mean FFM, whereas the remaining 9 equations [32,33,34,35,36,37,38,39,40] significantly overestimated FFM.

In Figure 1b, the trend line has a non-zero slope, which indicates so-called proportional bias. The mean difference in FFM estimates was greater in individuals with a high FFM. In some individuals with a high FFM, BIA seemed to overestimate FFM.

From these results, it is clear that none of the current available prediction equations can adequately predict the FFM DXA for this sample. Not one of the equations can predict FFM_DXA_ for this sample accurately up to 70%. This analysis in particular follows in the style of Marra et al. [21] and offers further confirmation that a new equation should be developed for this underrepresented sample in the literature.

### 3.2. Development of a New BIA Equation

Our final model retained sex, weight, reactance, and resistance index as significant predictors of FFM. The stepwise regression analysis yielded the following FFM prediction equation for this black preadolescent population:

FFM=105.20+0.807×Sex+0.174×Weight+0.01×Reactance+15.71×log(RI)

where weight is measured in kg, sex is coded as (0 – female, 1 – male). This model yielded R^2^ = 0.9544, and C (P) = 5.0. Residual analysis of the fitted model indicated that residuals were normally distributed (*p* > 0.05).

### 3.3. Cross-Validation of the New BIA Equation

The following approach was utilized: The sample was split into training and validation samples according to a 70:30 ratio; thus for the training sample *n* = 59, and the test sample *n* = 24. A multiple regression equation using the same identified variables as for the whole sample was estimated, using the training sample. Using the validation sample, the predicted FFM was calculated with the estimated multiple regression equations. The correlation between the true FFM and predicted FFM was then determined.

This process was repeated 5 times (similar to 5-fold cross validation), and in each instance the correlations where (i) high and (ii) highly significant; and the multiple regression equations for each of the 5 “folds” were observed to be similar with regards to magnitude of the estimated coefficients. Furthermore, in each case, all coefficients were found to be significant (*p* < 0.01). The results are contained in Table 4. To this effect, the authors are of the opinion that the multiple regression equation for the entire sample as proposed is reliable—especially for use in practice.

The new BIA equation yielded FFM values similar to FFM_DXA_ (*p* = 1). The new equation emerged as an excellent fit to the data.

## 4. Discussion

We present a new equation that can be used to estimate FFM in young, preadolescent, black children living in urban areas in South Africa. In our sample, 41% of the children were overweight indicating that there is a need to monitor obesity and body composition amongst this population group where obesity is expected to increase, resulting in significant co-morbidities in school-age children.

Monitoring body composition is an important part of monitoring nutritional status and clinical outcomes in adults and children. With the advent of new technology, BIA has become an important supporting tool for health professionals [11,12]. Most regression equations converting impedance-related data to body composition were developed for populations of a specific age, sex, race/ethnicity, or physical activity level [10,41,42] or for those suffering from particular diseases [26,43]. In our study, we examined the agreement of published BIA equations for predicting FFM by comparing the predicted values to values measured by DXA, the reference method. We subsequently developed a new equation and cross-validated the equation.

In our study, only two of the 17 prediction equations for calculating FFM in children showed statistically acceptable agreement with the FFM measured by DXA in pre-pubertal black South African children. Harchaoui et al. [44] found similar results for Moroccan children. Comparing FFM predicted from seven previously published equations to reference values for 247, eight to eleven-year-old children, they found that there was significant bias in predicted values [44]. The poor agreement between the predicted and measured values could be attributed to the population-specificity of published prediction equations. In a cross-sectional study on 864, eight to ten-year-old children from different ethnic groups, Nightingale et al. [45] found that generic equations used to estimate FFM, significantly under- or overestimated FFM, suggesting that ethnic- and sex specific BIA equations are needed. In this study, of the 17 equations that we investigated, only six were sex specific. Three of the sex specific equations [24,25,26] yielded FFM values that were similar to the reference FFM values measured by DXA suggesting that sex-specific equations may better estimate body composition, compared to equations that do not differentiate between sexes.

The measurements in this study were taken in the upright (standing) position, whereas in most previous studies participants were in the supine position. Thus, the comparisons (Table 3) are to be interpreted cautiously, as not only total body water but also water re-distribution within the body by a change of body position may affect bioelectrical impedance values [46].

The equations used in BIA are based on assumptions relating to the level of hydration, the fat fraction, and body shape and size [15]. In humans, the allometric relationships between trunk size and limb length differ across age and population groups resulting in the need for population specific BIA equations [13]. Men and women of different ages also have different body composition [45], and in the case of pubertal boys and girls, these differences are driven by changes in gonadal sex steroids [47]. In a cross-sectional study, Kirchengast [48] assessed the sex differences in body composition in 1649 children, aged six to 18 years. They found that girls had significantly more body fat and that boys had significantly higher lean body mass (LBM). Taylor et al. [49] assessed differences in body composition of boys and girls of similar age, height, weight, and BMI and also found that boys had significantly less body fat and higher LBM than girls. Our findings confirm sex differences in body composition and indicate that these differences may appear before puberty starts, highlighting the importance of specific equations for boys and girls of different ages.

Of the 17 equations that we tested, three equations predicted FFM values similar to our FFM reference values (*t*-test *p*-value > 0.05). These equations were all developed for children younger than 15 years and of white ethnicity. Although these equations yielded predictions close to observed values, there was limited agreement between measured and predicted FFM. Given that ethnicity influences body composition of children [12], we developed and cross-validated a specific equation for black children between the ages of five and eleven years attending school in an urban area. This equation would be more accurate and specific to black, preadolescent school aged children in South Africa. Our BIA equation resulted in a R^2^ values comparable to previous studies [25,27,28,29,39,40] and can be used to monitor body composition of healthy, black, preadolescent South African children.

Our findings may have limitations. We only had contact with the participants in the afternoon of the day with no control over the effect of hydration on BIA measurements. We asked children to avoid excessive physical activity 24 h before measurements, to void their bladder before measurement and to remain fasted for 2 h before measurement, except for the drinking yoghurt provided for comfort. In children, these factors are difficult to monitor. This study was also limited because we defined preadolescence in terms of age only. Our equation is thus only applicable for use in black, South African children aged 5–10 years. We performed cross-validation of the newly developed equation on the same data set from which the equation was developed, similar to Nielsen et al. [26]. Despite these limitations, our sample size is comparable to previously published validation studies on methods of assessing body composition [24,28,31].

Our study’s main value is the focus on the younger population. DXA remains the reference method for assessing body composition. Applying our equation in the BIA-based assessment of 5–10 year-old black South African children may result in practical and acceptable prediction of FFM.

## 5. Conclusions

In this paper, we show that most of the published BIA equations would not be suitable for use in South African children, aged between five and ten, of black ethnicity. We developed a new equation specifically for this population group, but there is still limited information on body composition techniques in children and adolescents in South Africa [13]. Future studies should keep this in mind, and we recommend that this equation be validated in other populations and the sample expanded before it is used to estimate FFM using BIA.

## Figures and Tables

**Table 1:** Summary of stepwise selection.

Step	Variable Entered	Partial R-Square	Model R-Square	C(p)	F Value	*p* Value
1	heightvar2	0.8907	0.8907	107.935	660.34	<0.0001
2	Weightvalue	0.0395	0.9302	42.3624	45.29	<0.0001
3	Reactance_Xc__50kHz_value	0.0183	0.9485	13.031	28.12	<0.0001
4	Sex	0.0059	0.9544	5	10.03	0.0022

**Table 2:** Participant characteristics and body composition estimates for total group and comparison between sex.

Parameters	Total (*n* = 84)	Boys (*n* = 40)	Girls (*n* = 44)	*p* Value
Age (y)	8.5 ± 1.4 ^1^	8.6 ± 1.5 ^1^	8.5 ± 1.4 ^1^	0.606
Weight (kg)	33.7 ± 10.9 ^1^	33.8 ± 11.0 ^1^	33.7 ± 11.0 ^1^	0.971
Height (cm)	132.2 ± 9.4 ^1^	132.6 ± 9.5 ^1^	131.8 ± 9.4 ^1^	0.711
HFAz	0.4 ± 1.0 ^1^	0.4 ± 1.0 ^1^	0.4 ± 1.0 ^1^	0.815
FM (kg) by DXA	11.8 ± 7.1 ^1^	10.6 ± 7.0 ^1^	13.0 ± 7.0 ^1^	0.127
FFM (kg) by DXA	21.5 ± 4.9 ^1^	22.8 ± 4.8 ^1^	20.4 ± 4.7 ^1^	0.025
Body fat % by DXA	33.4 ± 9.7 ^1^	29.5 ± 9.4 ^1^	36.8 ± 8.7 ^1^	<0.001
BMIFA*z*	1.0 ± 1.6 ^1^	1.1 ± 1.8 ^1^	1.0 ± 1.5 ^1^	0.824
**BMI-for-Age Classification**				
−2 > and ≤ 1 z-score	49 (59) ^2^	24 (62) ^2^	25 (57) ^2^	
> 1 z-score	34 (41) ^2^	15 (38) ^2^	19 (43) ^2^	
**BIA Values**				
Reactance (Ω)	65.3 ± 8.5 ^1^	64.5 ± 8.1	65.2 ± 8.7	
Resistance (Ω)	815.1 ± 98.4 ^1^	775.9 ± 87.1	845.0 ± 91.0	
Impedance (Ω)	817.8 ± 98.6 ^1^	778.6 ± 87.3	847.6 ± 91.2	
Resistance index (cm^2^/ Ω)	21.9 ± 5.0 ^1^	23.5 ± 5.5	20.8 ± 4.5	
Impedance index (cm^2^/ Ω)	21.8 ± 5.0 ^1^	23.4 ± 5.2	20.8 ± 4.5	

^1^ mean ± SD; ^2^ number (column percentage). HFAz: Height-for-age z-score; DXA: dual-energy X-ray absorptiometry; BMIFAz: BMI-for-age z-score; FM: fat mass; FFM: fat-free mass; BMI: body mass index; BIA: Bioelectrical impedance analysis.

**Table 3:** Agreement between participants FFM measured by DXA versus FFM predicted by BIA equations.

	FFM (kg) ^1^	Mean Difference (kg) ^1^	Bias % ^2^	Prediction % Accurate ^3^; under ^4^; over ^5^	*p*-Value ^6^	Lower and Upper LOA	ICC	Power Calculation ^7^
**Measured DXA (FFM_DXA_)**	20.5 ± 4.9 ^1^							
**Predicted with Equation (FFM_BIA_)**								
Clasey et al. [24]	21.7 ± 5.0 ^1^	−0.2 ± 1.3 ^1^	−1.0	14.3; 34.5; 51.2	0.269	−2.68; 2.37	0.97 (0.95; 0.98)	0.196
Deurenberg et al. [25]	21.6 ± 5.9 ^1^	−0.1 ± 1.7^1^	−0.5	13.1; 41.7; 45.2	0.680	−3.47; 3.32	0.95 (0.93; 0.97)	0.069
Nielsen et al. [26]	21.5 ± 4.0 ^1^	−0.0 ± 1.3 ^1^	0	16.7; 35.7; 47.6	0.954	−2.68; 2.66	0.95 (0.94; 0.97)	0.05
Bedogni et al. [27]	20.2 ± 3.5 ^1^	1.3 ± 1.9 ^1^	+6.3	27.4; 44.0; 28.6	<0.001	−2.5; 5.03	0.86 (0.80; 0.92)	>0.999
Charatsi et al. [28]	20.3 ± 5.5 ^1^	1.2 ± 1.4 ^1^	+5.9	11.9; 48.8; 39.3	<0.001	−1.60; 3.97	0.94 (0.91; 0.96)	>0.999
Pietrobelli et al. [29]	20.1 ± 3.3 ^1^	1.4 ± 2.1 ^1^	+6.8	29.8; 41.7; 28.6	<0.001	−2.78; 5.50	0.83 (0.76; 0.90)	>0.999
Schaefer et al. [30]	20.3 ± 3.9 ^1^	1.2 ± 1.8 ^1^	+5.9	19.0; 41.7; 39.3	<0.001	−2.44; 4.78	0.88 (0.84; 0.93)	>0.999
Tyrrell et al. [31]	19.4 ± 4.1 ^1^	2.1 ± 1.3 ^1^	+10.2	17.9; 53.6; 28.6	<0.001	−0.49; 4.61	0.86 (0.81; 0.92)	>0.999
Cordain et al. [32]	24.8 ± 4.0 ^1^	−3.3 ± 1.6^1^	−16.1	9.5; 11.9; 78.6	<0.001	−6.41; −0.09	0.71 (0.60; 0.81)	>0.999
De Lorenzo et al. [33]	22.5 ± 5.0 ^1^	−1.0 ± 1.3^1^	−4.9	17.9; 31.0; 51.2	<0.001	−3.58; 1.67	0.95 (0.92; 0.97)	>0.999
Deurenberg et al. [34]	22.3 ± 6.1 ^1^	−0.8 ± 2.0^1^	−3.9	8.3; 42.9; 48.8	0.001	−4.89; 3.29	0.92 (0.89; 0.95)	0.942
Duncan et al. [35]	22.9 ± 5.9 ^1^	−1.4 ± 2.0^1^	−6.8	15.5; 32.1; 52.4	<0.001	−5.38; 2.53	0.90 (0.86; 0.94)	>0.999
Eston et al. [36]	24.2 ± 5.3 ^1^	−2.7 ± 1.6^1^	−13.2	20.2; 17.9; 61.9	<0.001	−5.89; 0.50	0.83 (0.76; 0.89)	>0.999
Horlick et al. [37]	23.9 ± 5.0 ^1^	−2.4 ± 1.5^1^	−11.7	13.1; 21.4; 65.5	<0.001	−5.39; 0.51	0.84 (0.78; 0.91)	>0.999
Houtkooper et al. [38]	23.2 ± 5.5 ^1^	−1.7 ± 1.5^1^	−8.3	17.9; 27.4; 54.8	<0.001	−4.82; 1.34	0.90 (0.86; 0.94)	>0.999
Houtkooper et al. [39]	23.6 ± 5.2 ^1^	−2.1 ± 1.4^1^	−10.2	17.9; 22.6; 59.5	<0.001	−4.99; 0.76	0.88 (0.83; 0.93)	>0.999
Rush et al. [40]	22.8 ± 5.4 ^1^	−1.3 ± 1.5^1^	−6.3	16.7; 31.0; 52.4	<0.001	−4.24; 1.60	0.93 (0.90; 0.96)	>0.999

^1^ mean ± SD. ^2^ Bias = mean percentage difference between FFM_BIA_ and FFM_DXA_. ^3^ Accurate prediction = percentage of FFM_BIA_ values within ±5% of FFM_DXA_. ^4^ Underprediction = percentage of FFM_BIA_ values < 5% of FFM_DXA_. ^5^ Overprediction = percentage of FFM_BIA_ values >5% of FFM_DXA_. ^6^ Paired *t*-test; ^7^ *P*-value of a two-sample (dependent) paired *t*-test; FFM: fat-free mass; LOA: limits of agreement; ICC: intra-class correlation coefficient.

**Table 4:** Cross-validation process of new BIA equation.

Fold	Predicted Equation Using Training Sample	Adjusted R Square of Equation	Correlation Coefficient between Estimated FFM Using Validation Sample and True FFM
1	105.08013 + 0.72964 × Sex + 0.18292 × Weight + 0.09597 × Reactance + 15.68951 × log(RI)	0.9491	0.96844
2	102.32036 + 0.85808 × Sex + 0.17076 × Weight + 0.07864 × Reactance + 15.01190 × log(RI)	0.9574	0.96832
3	99.63712 + 0.98731 × Sex + 0.17902 × Weight + 0.07445 × Reactance + 14.56929 × log(RI)	0.9578	0.96844
4	104.99849 + 0.87828 × Sex + 0.18278 × Weight + 0.09955 × Reactance + 15.71988 × log(RI)	0.9498	0.96951
5	103.54027 + 0.63764 × Sex + 0.17154 × Weight + 0.08310 × Reactance + 15.24419 × log(RI)	0.9586	0.96717
